# Metabolomic and Transcriptomic Analyses Reveal the Molecular Mechanism Underlying the Massive Accumulation of Secondary Metabolites in Fenugreek *(Trigonella foenum-graecum* L.) Seeds

**DOI:** 10.3390/genes15030343

**Published:** 2024-03-07

**Authors:** Qiuyu Zhao, Guoxing Wu, Pu Yang, Yuanchong Shi, Zuoyi Fu, Haifeng Mo, Chunlan Shi, Shuhui Yu

**Affiliations:** 1 College of Agriculture and Life Sciences, Kunming University, Kunming 650214, China; zqy19951010@163.com; 2 College of Plant Protection, Yunnan Agricultural University, Kunming 650201, China; wugx1@163.com (G.W.); shiclan22@163.com (C.S.); 3 Institute of Highland Forest Science, Chinese Academy of Forestry, Kunming 650224, China; zjuyangpu@aliyun.com (P.Y.); cherub11520@163.com (Y.S.); fuzuoyi000@yeah.net (Z.F.); mohf18@lzu.edu.cn (H.M.); 4 Key Laboratory of Breeding and Utilization of Resource Insects of National Forestry and Grassland Administration, Kunming 650224, China

**Keywords:** fenugreek seeds, metabolites, metabolome, flavonoids, transcriptome, antifungal activity, *Alternaria tenuissima*, *Magnaporthe oryzae*

## Abstract

Fenugreek *(Trigonella foenum-graecum* L.) is a traditional medicinal plant for treating human diseases that is widely cultivated in many countries. However, the component and related metabolic pathways are still unclear. To understand the changes in expression of the component and related genes during seed development, this study employed metabolomic and transcriptomic analyses and integrative analysis to explore the metabolites and pathways involved in the growth of fenugreek. The antifungal activity of the fenugreek seeds was also analyzed. A total of 9499 metabolites were identified in the positive ion mode, and 8043 metabolites were identified in the negative ion mode. Among them, the main components were fatty acyls, prenol lipids, steroids, steroid derivatives, flavonoids, and isoflavonoids. Among these enriched pathways, the top 20 pathways were “flavone and flavonol biosynthesis”, “isoflavonoid biosynthesis”, and “flavonoid biosynthesis”. 3,7-Di-O-methylquercetin, flavonoids, pseudobaptigenin, isoflavonoids, methylecgonine, alkaloids, and derivatives were the most significantly upregulated metabolites. There were 38,137 differentially expressed genes (DEGs) identified via transcriptomic analysis. According to the KEGG pathway enrichment analysis, 147 DEGs were significantly enriched in “flavonoid biosynthesis”. Ten DEGs of the six key enzymes were found to be involved in three pathways related to flavonoid and alkaloid synthesis in fenugreek. The antifungal activity test revealed the inhibitory effect of the ethanol extract of fenugreek seeds on *Alternaria tenuissima (Kunze)Wiltshire* and *Magnaporthe oryzae*. These findings further prove that the use of botanical pesticides in fenugreek fruit has research value.

## 1. Introduction

Fenugreek *(T. foenum-graecum* L.) is an annual herbaceous plant of the Fabaceae and subfamily Papilionaceae. The plant has pale yellow triangular flowers and triangular leaves. It has strong adaptability to the environment because it is shade-loving, cold-tolerant, and drought-tolerant [1]. Fenugreek was first found in Iran and northern India, and it was first introduced to China during the Song dynasty as a spice [2]. Currently, it is widely planted in Asia, Africa, and Mediterranean countries. India is the main producer of fenugreek [3].

Fenugreek has been used in many fields, such as medicine, perfume, cosmetics, and livestock feed. Fenugreek is an important medicinal plant in traditional medicine in many countries [4,5]. Fenugreek has many well-known pharmacological effects, including treating hypoglycemia and hypocholesterolemia; gastroprotective, antioxidant, anti-inflammatory, and antipyretic activities; and appetite stimulation [4,5]. In ancient Egypt, fenugreek was used to improve the milk production of breastfeeding mothers. It has also been used as a folk medicine to treat cellulitis, bacteria, and tuberculosis. In the 19th century, fenugreek was still the main component of medicine used to treat dysmenorrhea [6]. Fenugreek is also widely used in the field of traditional Chinese medicine. The seeds of fenugreek plants are used in traditional Chinese medicine to clear congestion and treat lung disease [7].

Currently, people are paying more attention to the new medicinal potential of fenugreek. Some studies have shown that fenugreek surgery has favorable effects on rheumatoid arthritis [8]. The ethanolic extract of fenugreek has an inhibitory effect on the deleterious phenomenon of glycation, and it can be a nutraceutical against aging and associated morbidities [9]. The hydroalcoholic extract of fenugreek seeds has an inhibitory effect on the gene expression and enzymatic activity of several important factors (bFGF, VEGF, and MMP-2/MMP-9) involved in angiogenesis [10]. Fenugreek methanolic extracts suppressed the metastasis and proliferation of MCF-7 breast cancer cells [11]. Thus, fenugreek might be a good resource for exploring compounds with anticancer properties, and it is necessary to pay attention to the components of fenugreek.

A study revealed that the seeds of fenugreek plants contain many alkaloids (such as trigonelline, gentianine, and carpaine) and flavonoids (such as rutin, quercetin, and vitexin) [7]. These metabolites may have pharmacological effects on human diseases. It has been found that trigonelline, diosgenin, and 4-hydroxyisoleucine have neuroprotective effects on Alzheimer’s disease [3]. These compounds have antibacterial, anti-inflammatory, anticancer, antiviral, antioxidant, hypoglycemic, etc., activities [12,13]. One study found that trigonelline helps to reduce diabetic nephropathy in rats [12]. Another study showed that vitexin can suppress renal cell carcinoma [13]. Therefore, the components of fenugreek are thought to have therapeutic effects on human disease. However, the current relevant research still lacks global knowledge of the secondary metabolites of fenugreek. In addition, there is little research on the accumulation of secondary metabolites during the growth of fenugreek seeds.

In recent years, transcriptome sequencing of fenugreek tissues from leaves, stems, and roots has been conducted. The transcripts may be responsible for the coding of enzymes involved in the biosynthesis of trigonelline, diosgenin, 4-hydroxyisoleucine, quercetin, and other secondary metabolites in fenugreek [14]. However, there is little research on the mechanism of secondary metabolite synthesis in fenugreek seeds. In addition, fenugreek may have antifungal or antibacterial effects. Several studies have shown that fenugreek seeds have inhibitory effects on several drug-resistant bacteria [15]. However, studies on the inhibitory effects of the component of fenugreek on phytopathogenic fungi are rare.

To identify the important accumulated metabolites and the key genes involved in these secondary metabolites, transcriptome sequencing, metabolome analyses, and transcriptome–metabolome integrative analysis were subsequently conducted on the seeds of fenugreek plants. In addition, antibacterial activity analysis was conducted on the ethanol extracts of fenugreek seeds to further determine the value of botanical pesticides in treating these plants. These analyses will provide new information on the substance and gene expression changes that occur during fenugreek seed development and will also broaden the utilization of fenugreek seeds.

## 2. Materials and Methods

### 2.1. Plant Materials

Fenugreek plants (*T. foenum-graecum*) were collected from the Institute of Highland Forest Science of the Chinese Academy of Forestry in Kunming, Yunnan Province, China. The seeds of fenugreek in early and late developmental stages were selected as the experimental materials. The seeds were rapidly frozen in liquid nitrogen for further metabolome and transcriptome analyses. Three replicates were used for transcriptome analyses, and six replicates were used for metabolome analysis.

### 2.2. Plant Pathogenic Microorganism Materials

Rice blast fungus (*Magnaporthe oryzae*) and tobacco brown spot (*Alternaria tenuissima (Kunze)Wiltshire*) were obtained from Dr. Guo-Xing Wu of the College of Plant Protection, Yunnan Agricultural University.

### 2.3. Metabolomic Analysis

First, the metabolites were extracted from the seeds by the protein precipitation method with organic reagents. Briefly, the seed samples were weighed accurately to 25 mg and put into 1.5 mL centrifuge tubes. A total of 800 μL of precooled precipitant solution (methanol/acetonitrile/pure water = 2:2:1) was added to the tube. Then, two steel balls were used to grind the samples in a grinder at 60 Hz. The samples were ground for 4 min, followed by ultrasonication in a water bath at 80 Hz for 10 min. Then, the samples were put into a refrigerator at −20 °C for 120 min. Subsequently, the samples were centrifuged at 25,000× *g* and 4 °C for 15 min. Approximately 600 µL of the supernatant was obtained, and the procedure was repeated again. The supernatant was subsequently drained in a freeze dryer. Then, 600 µL of 10% methanol was added, and the mixture was subjected to water bath ultrasonication at 80 Hz for 10 min. Subsequently, the samples were centrifuged at 25,000× *g* and 4 °C for 15 min. Then, the supernatant was obtained. Finally, 50 µL of each sample was extracted and mixed for quality control.

### 2.4. Liquid Chromatography–Mass Spectrometry (LC–MS)

All of the samples were analyzed by an LC–MS system. The ACQUITY UPLC HSS T3 column (100 mm × 2.1 mm, 1.8 μm; Waters, Wilmslow, UK) was used to conduct chromatographic separation. The column was maintained at 50 °C. The 0.4 mL/min flow rate was set. Mobile phase A was water and 0.1% formic acid. Mobile phase B was methanol and 0.1% formic acid. The metabolite was subsequently eluted. There was 5 µL sample was loaded. The metabolites eluted from the column which were detected by Xevo G2 XS QTOF high-resolution tandem mass spectrometer (Waters, UK). The small molecules that eluted from the column were extracted in both positive and negative ion modes by Q-TOF. The data were subsequently collected in Centroid MSE mode. Furthermore, forevaluating the stability of the LC–MS during the whole collection process, ten quality control samples were used to balance the instrument, and then one quality control sample was interspersed during the test. Finally, three quality control samples were loaded.

### 2.5. Metabolic Information Analysis

First, the peak list information was extracted from the raw data, and correction was subsequently conducted. Peak extraction was achieved mainly by using Progenesis QI (version 2.2). The differentially abundant metabolites were identified via a series of statistical analyses, and a fold change greater than or equal to 1.2 or less than or equal to 0.8333 and a q value less than 0.05 were used as the thresholds for screening differentially abundant metabolites. Finally, the differentially abundant metabolites were identified, and enrichment analysis of the pathways was conducted via the Kyoto Encyclopedia of Genes and Genomes (KEGG) database.

### 2.6. Library Construction and Sequencing

Total RNA was extracted from frozen seeds according to the Trizol reagent (Invitrogen, Waltham, MA, USA), and mRNA fragments were obtained via the mRNA enrichment method or the rRNA depletion method. For the mRNA enrichment method, mRNAs with poly(A) tails were enriched from total RNA by magnetic beads with OligodT. For the rRNA depletion method, DNA was probed to hybridize rRNA, the DNA/RNA hybrid strands were selectively digested by RNase H, the DNA was digested by DNase I, and the mRNA was subsequently obtained after purification. Then, the RNA was fragmented with interrupt buffer, and the random N6 primer was used to perform reverse transcription. Finally, double-stranded cDNA was obtained after reverse transcription. Then, the synthesized double-stranded DNA was filled in. The 5′ end was phosphorylated. It was connected with the bubble connector such that the 3′ end had a base “T”. Then, the products were subjected to PCR amplification. Subsequently, the PCR products formed single-stranded DNA after thermal denaturation, and the single-stranded circular DNA was extracted by a bridge primer. The library was subsequently sequenced on the BGISEQ-500 platform.

### 2.7. Data Filtering

The data were analyzed using SOAPnuke (v1.4.0) and filtered using Trimmomatic (v0.36). The clean reads were obtained after removing the reads with linker contamination, reads with an unknown base N content greater than 5%, and low-quality reads.

### 2.8. De Novo Assembly

The clean reads were de novo-assembled by Trinity (v2.0.6) [16]. The transcripts were clustered, and redundancies were removed via CD-HIT (v4.6). Then, the unigenes were obtained. BUSCO was subsequently used to assess the quality of the assembled transcripts. To some extent, the integrity of the assembled transcripts can be assessed via comparison with conserved genes.

### 2.9. Coding Sequence (CDS) Prediction

Transdecoder (v3.0.1) was used to identify the candidate coding regions. First, the longest open-reading frame was extracted, and the unigene.fa file was aligned with the SwissProt database using Diamond BLASTP based on sequence similarity. The Pfam protein homologous sequence was subsequently searched via BLAST, after which the sequences were compared with those of SwissProt and Hmmscan. Subsequently, the CDS was predicted by Transdecoder (v3.0.1).

### 2.10. Gene Annotation

The assembled unigenes were annotated through BLAST against seven functional databases: KEGG, Gene Ontology (GO), the Nonredundant Protein Sequence Database (NR), the Nucleotide Sequence Database (NT), SwissProt, Protein Family Analysis and Modeling (Pfam), and Clusters of Orthologous Groups for Eukaryotic Complete Genomes (KOG).

### 2.11. Gene Quantification

All the reads from the early and late developmental samples were mixed and assembled to construct a transcriptome. The reads of each sample were compared with the transcripts of the corresponding transcriptome for quantitative analysis. Bowtie2 software (v2.2.5) was used to align the clean reads to the assembled reference sequences via de novo assembly, and the gene expression levels in each sample were evaluated via RSEM (v1.2.8) [17]. In R, PCA was performed using the princomp function in R and a correlation matrix. Further quality control was performed using boxplots, histograms, density plots, and Venn diagrams. The genes were clustered according to their similar expression profiles by Mfuzz v2.34.0. The genes with consistent expression trends may be involved in similar biological processes.

### 2.12. Differentially Expressed Gene (DEG) Analyses

Finally, differential expression analysis was performed according to the clean reads. The differentially expressed genes (DEGs) were identified by using the DEGseq method based on the Poisson distribution [18]. The significantly differentially expressed genes were identified by comparing the late group with the early group. The screening criteria were a fold change greater than or equal to 2 and an adjusted p value less than or equal to 0.001. The DEGs were subsequently obtained.

GO enrichment analyses of the DEGs were subsequently performed. The GO terms were divided into molecular function, cellular component, and biological process. Moreover, the phyper function in R (https://en.wikipedia.org/wiki/, accessed on 24 October 2019) was used to adjust the p-value according to the FDR. Usually, a q-value less than or equal to 0.05 was regarded as indicating statistical enrichment.

KEGG enrichment analyses were subsequently carried out. The classifications included cellular processes, environmental information processing, genetic information processing, human disease, metabolism, organismal systems, and drug development. The enrichment analysis method was the same as that used for GO enrichment.

### 2.13. Integrated Analysis of the Transcriptome and Metabolome

First, the significantly upregulated metabolites were filtered out of the metabolome analysis. The filtering process was same as the screening of differential metabolites in metabolome analysis. The differentially expressed genes were filtered according to log2(fold change) > 9 or log2(fold change) < −9 in the transcriptome. The DEGs were compared to the metabolic pathways by Pearson’s Correlation Analysis. Finally, the key regulatory genes which correlated with the significantly upregulated metabolite synthesis were analyzed.

### 2.14. Preparation of the Crude Ethanol Extract of Fenugreek Seeds

First, the fenugreek seeds were ground with an electric grinder (LINGSUM, Lishui, Zhejiang, China), and the powder was filtered through a 60-mesh sieve. Then, 800 mL of 95% ethanol was added to 80 g of powder from the fenugreek seeds in a 1000 mL Erlenmeyer flask, after which the mixture was soaked for 12 h. Then, the powder mixture was ultrasonicated by an ultrasonic homogenizer (JY92-IIN, SCIENTZ, Ningbo, Zhejiang, China). The solution was extracted by removing the precipitate. The extraction solid–liquid ratio was 1:10. Then, 800 mL of 95% ethanol was added to the Erlenmeyer flask, and the extraction process was repeated. Finally, the ethanol extract of the fenugreek seeds was obtained by 54 °C rotary evaporation (NVC-2000, EYELA, Tokyo, Japan).

### 2.15. Antifungal Activity Test of Fenugreek Seed Extracts

First, the ethanol extract of the fenugreek seeds was dissolved in 1% DMSO, and the stock solution was diluted to 2 g/mL. Then, 1 mL of mother liquor was added to 100 mL of potato dextrose agar (PDA) medium and mixed completely. The final concentration was 20 mg/mL. The medium was poured into 9 cm petri dishes as the late group culture medium. An equal amount of DMSO (1%) was added to the CK group, and the blank control group was PDA medium. Two pathogenic fungi (*Alternaria tenuissima (Kunze)Wiltshire* and *Magnaporthe oryzae*) were selected for this test. The pathogenic fungi were cultivated for two generations and the fungal aliquots were obtained by the hole-punch method. All the treatment groups, CK groups, and blank controls had three repetitions. The plants were cultivated in an incubator at 27 ± 0.5 °C in a dark environment for 7 days. The growth of the pathogenic fungi, including their diameter, density, and color, was recorded every day. The diameter of the pathogenic fungi was measured by the cross-bonded method when the pathogenic fungi in the blank group had grown to the edge of the medium. The statistical analysis of diameters was performed by one-way ANOVA test in SPSS. Finally, the antifungal rate in each group was obtained. These parameters were calculated according to the following formula:

Antifungal rate = (1 − (experiment colony diameter/CK colony diameter)) × 100%.

## 3. Results

### 3.1. Fenugreek Metabolome Analysis

**Figure 1:**
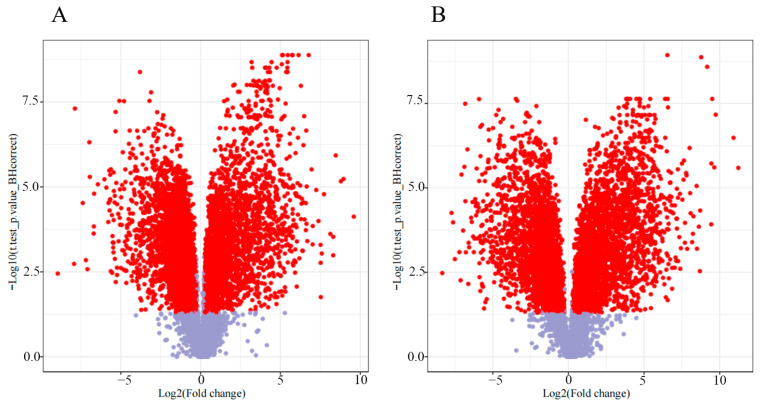
The volcano maps of metabolites in the early and late seed developmental stages in positive ion mode and negative ion mode. Red dots represent significant different metabolites, and gray dots represent non-significant different metabolites. (**A**) Volcano map in positive ion mode; (**B**) volcano map in negative ion mode.

A total of 9499 metabolites were identified in the positive ion mode, and 8043 metabolites were identified in the negative ion mode. After comparing the metabolites between early and late development stages, a total of 2848 differentially abundant metabolites were identified via the positive ion mode, and 2625 differentially abundant metabolites were identified via the negative ion mode. There were 1415 metabolites upregulated and 1433 metabolites downregulated in the positive ion mode. A total of 1450 metabolites were upregulated, and 1175 metabolites were downregulated according to the negative model (Figure 1). This figure was drawn based on the data from Table S1.

**Figure 2:**
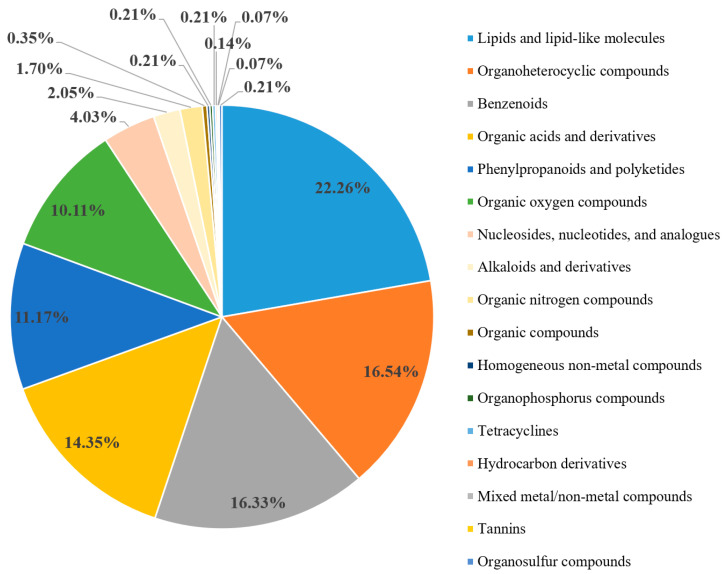
Pie chart of secondary metabolites detected by the metabolomic analysis.

There were 315 “lipids and lipid-like molecules”, 234 “organoheterocyclic compounds”, 231 “benzenoids”, 203 “organic acids and derivatives”, 158 “phenylpropanoids and polyketides”, 143 “organic oxygen compounds”, 57 “nucleosides, nucleotides”, and “analogs”, and 29 “alkaloids and derivatives” among these metabolites (Figure 2).

In addition, the metabolites “benzenoids”, “organic acids and derivatives”, “phenylpropanoids and polyketides”, and “alkaloids and derivatives” also accounted for an important proportion of the composition. Among the “phenylpropanoids and polyketides”, 58 were “flavonoids” and 16 were “isoflavonoids”. Among the “benzenoids”, there were 151 “benzene and substituted derivatives”. Among the alkaloids and their derivatives, 5 were “camptothecins”, 3 were “morphinans”, and 3 were “tropane alkaloids” (Figure 2 and Figure S1). These figures were drawn based on the data from Tables S1 and S2. 

### 3.2. Principal Component Analysis (PCA)

**Figure 3:**
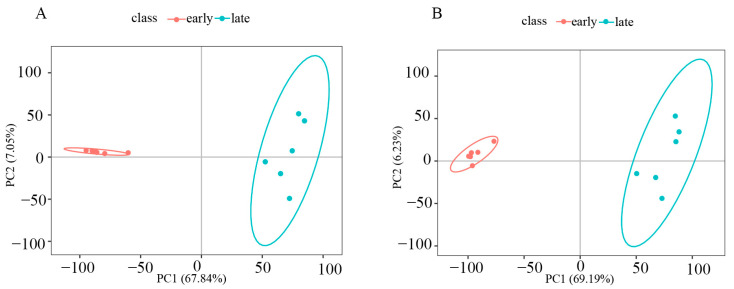
Principal component analysis of metabolites in the early and late seed developmental stages in positive ion mode and negative ion mode. (**A**) Principal component analysis in positive mode; (**B**) principal component analysis in negative mode.

The fenugreek early seeds were used as the early group for comparing metabolite changes with those of late seeds. The late group and early group were clustered in different quadrants. The seeds of fenugreek in the early and late stages of development were segregated on PC1 in both positive and negative ion modes. The PCA results showed that the metabolites of fenugreek seeds at different stages of development were significantly different (Figure 3). This figure was drawn based on the data from Table S1.

### 3.3. Cluster Heatmap Analysis

**Figure 4:**
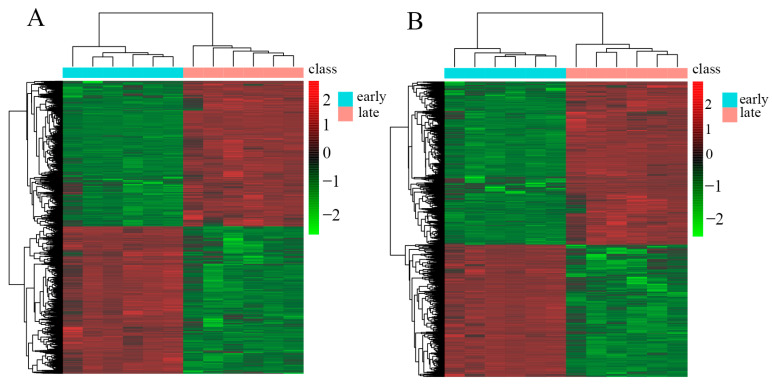
Correlation analysis diagram of differentially accumulated metabolites. (**A**) Differential ion cluster analysis in positive mode; (**B**) differential ion cluster analysis in negative mode.

A clustering heatmap was generated to examine the different metabolites between the early and late groups. Analysis of the cluster heatmap in positive and negative ion modes showed that the metabolites in the different groups were significantly different. The heatmap was divided into four main clusters: the metabolites involved in clusters 1 and 2 accumulated at the highest levels in the late group, and the metabolites involved in clusters 3 and 4 accumulated at the highest levels in the early group (Figure 4). These figures were drawn based on the data from Table S1. 

### 3.4. Enrichment Analyses of Differentially Accumulated Metabolites (DAMs) and Metabolites in Pathways Related to Flavonoids and Alkaloids

According to the KEGG enrichment analysis of the metabolites, 100 pathways were related to metabolism, which included 18 “biosynthesis of other secondary metabolites”, 14 “carbohydrate metabolism”, 14 “amino acid metabolism”, and 13 “lipid metabolism”.

**Figure 5:**
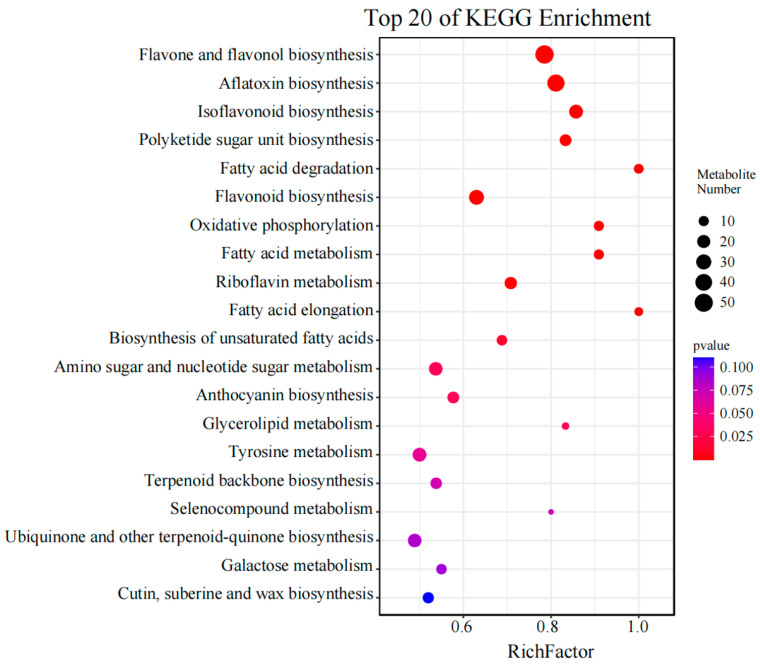
Bubble chart of KEGG pathway enrichment analysis for comparing the differentially accumulated metabolites between early and late seed developmental stages.

Among these enriched pathways, the top 20 pathways are shown in Figure 5. There were 51 metabolites significantly enriched in “flavone and flavonol biosynthesis” (ko00944), 24 metabolites significantly enriched in “isoflavonoid biosynthesis” (ko00943), and 29 metabolites significantly enriched in “flavonoid biosynthesis” (ko00941) (Figure 5). This figure was drawn based on data from Table S3.

According to the identification of metabolites, 16 flavonoids, 4 isoflavonoids, 2 alkaloids, and their derivatives were significantly upregulated in late-developmental-stage seeds. These metabolites were mostly enriched in pathways related to the syntheses of flavonoids, isoflavonoids, and alkaloids, such as “flavone and flavonol biosynthesis” (ko00944); “flavonoid biosynthesis” (ko00941); “isoflavonoid biosynthesis” (ko00943); “tropane, piperidine and pyridine alkaloid biosynthesis” (ko00960); and “isoquinoline alkaloid biosynthesis” (ko00950) (Table 1). This table was drawn based on data from Table S3.

**Figure 6:**
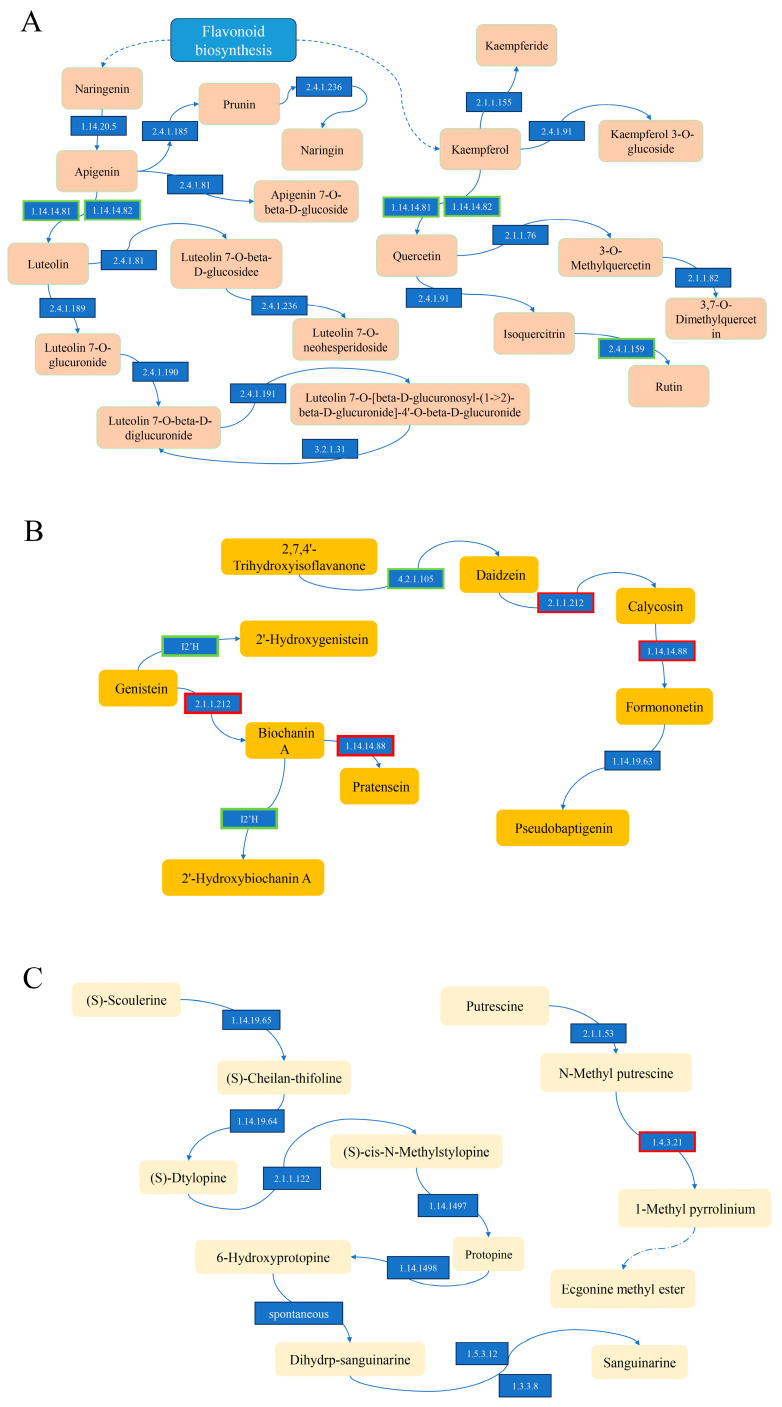
The speculative metabolic pathways of flavonoids, isoflavonoids, and alkaloids and derivatives in fenugreek. (**A**) The metabolic pathway of flavonoids; (**B**) the metabolic pathway of isoflavonoids; (**C**) the metabolic pathway of alkaloids and derivatives. The numbers in this figure are the EC number of enzymes. The arrow represents the direction of synthesis of the compound.

Among the flavonoids, 3,7-di-O-methylquercetin, luteolin 7-O-β-D-diglucuronide, apigenin, apigenin 7-O-β-D-glucoside, naringin, chrysoeriol, rutin, kaempferol 3-O-glucoside, luteolin 7-O-β-D-glucoside, dihydrokaempferol, luteolin 7-O-[β-D-glucuronosyl-(1->2)-β-D-glucuronide]-4′-O-β-D-glucuronide, epigallocatechin 3-gallate, phlorizin, kaempferide, (+)-catechin, and naringenin were the most significantly upregulated flavonoids (Table 1). The KEGG analysis showed that apigenin was synthesized from naringenin (Figure 6). This figure was drawn based on data from Table S3.

Pseudobaptigenin, 2′-hydroxybiochanin A, 2′-hydroxygenistein, and pratensein were the most significantly upregulated flavonoids in the synthesis of isoflavonoids. 3,7-Di-O-methylquercetin was stepwise synthesized from kaempferol, and pseudobaptigenin was synthesized from calycosin (Figure 6).

Among the synthesized alkaloids and derivatives, the ecgonine methyl ester and sanguinarine were the most significantly upregulated. Sanguinarine was gradually synthesized from (S)-scoulerine, and ecgonine methyl ester was gradually synthesized from putrescine (Figure 6).

### 3.5. Analysis of Fenugreek Seed Transcriptome Data

**Figure 7:**
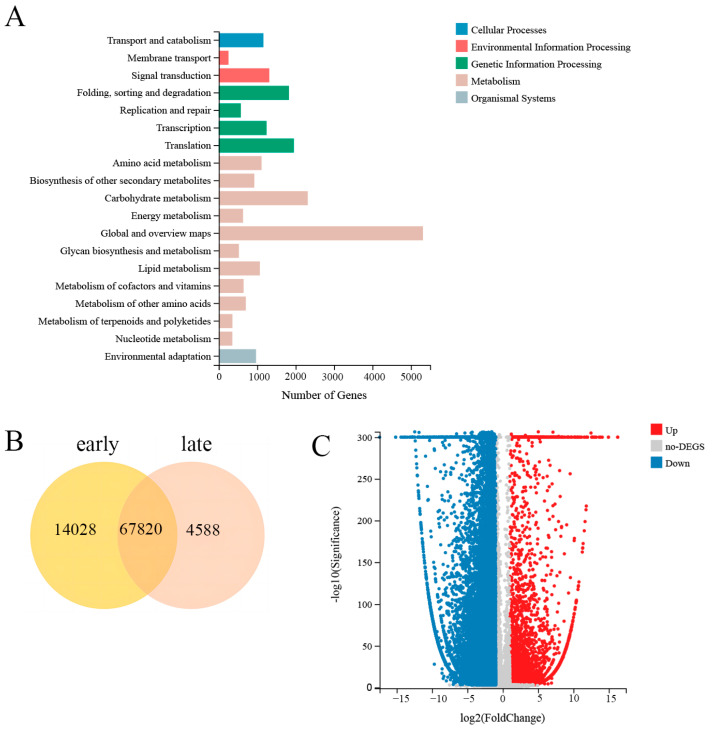
The results of transcriptome analysis of DEGs of fenugreek. (**A**) Venn diagram of differentially expressed genes between the early and late seed developmental stages; (**B**) volcano plot of DEGs between the early and late seed developmental stages; (**C**) the classification of KEGG pathways of differentially expressed genes between early and late seed developmental stages.

A total of 39.47 Gb of raw data were obtained from the six transcriptomic sequencing datasets. After assembly and removal of redundant sequences, 91,744 unigenes were obtained from the reads. Among these genes, 4588 unique genes were identified in the late group, 14,028 unique genes were identified in the early group, and 67,820 common genes were identified (Figure 7). The average length was 1316 bp, the N50 was 2119 bp, and the GC content was 38.12%. There were 60,633 (66.09%), 62,672 (68.31%), 42,458 (46.28%), 45,123 (49.18%), 45,995 (50.13%), 48,108 (52.44%), and 43,056 (46.93%) unigenes annotated in the following seven functional databases: NR, NT, SwissProt, KOG, KEGG, GO, and Pfam. Five functional databases were selected to construct a Venn diagram of the gene annotations (Figure S2).

### 3.6. Differential Expression Gene (DEG) Analysis

A total of 38,137 DEGs were identified, including 5233 upregulated genes and 32,904 downregulated genes. There were 14,242 common DEGs in the early vs. late groups (Figure 7). Figure S2 and Figure 7 and Table 2 were made based on the data from Figures S4 and S5.

### 3.7. KEGG and GO Enrichment Analysis of DEGs

The DEGs were assigned to the GO functional classification, and 32,313 DEGs were annotated into 18 subclasses of “biological processes”. Among them, the numbers of DEGs associated with “cellular processes” and “metabolic processes” were greatest, with 9120 and 7861 DEGs, respectively (Table S4).

KEGG enrichment analyses revealed that 6407 DEGs were annotated as “metabolic”. In addition to “amino acid metabolism”, “lipid metabolism”, and “carbohydrate metabolism”, “biosynthesis of other secondary metabolites” also accounted for the largest number of genes. There were 925 DEGs enriched in “biosynthesis of other secondary metabolites”. Among them, 147 DEGs were enriched in “flavonoid biosynthesis” (ko00941) (Table S5). 

### 3.8. Integrated Metabolome and Transcriptome Analysis of Flavonoids and Alkaloids

According to the results of the integrative analysis of the transcriptome and metabolome, 169 DEGs were significantly correlated with 344 DAMs (Figure S3). Among them, 9 flavonoids, 2 isoflavonoids, and 2 alkaloids and their derivatives were found to be significantly related to nine DEGs. These DEGs and corresponding DAMs were as follows. The legumin B gene was shown to be significantly related to the flavonoids apigenin, chrysosplenol D, and astragalin. Two genes (legumin J and seed linoleate 9S-lipoxygenase-3) were shown to be significantly related to resokaempherol among the flavonoids. Two genes (H/ACA ribonucleoprotein complex subunit 4 and HMG (high mobility group) box protein with ARID) were shown to be related to kaempferitrin in flavonoids (Table 2). According to the metabolome enrichment analysis, the biosynthesis of flavonoids and alkaloids was the main pathway involved. Moreover, three pathways were also identified via transcriptomic analyses. The three pathways were “tropane, piperidine and pyridine alkaloid biosynthesis” (ko00960), “flavone and flavonol biosynthesis” (ko00944), and “isoflavonoid biosynthesis” (ko00943). Among these pathways, 10 DEGs associated with six key enzymes were found to be involved in the synthesis. Table 2 and Figure S3 were made based on the data from Table S6.

Primary amine oxidase (EC:1.4.3.21, 1 gene) was found in “tropane, piperidine and pyridine alkaloid biosynthesis” (ko00960) and was upregulated during the synthesis of ecgonine methyl ester (Figure 6).

The flavanoid 3′,5′-hydroxylase (EC:1.14.14.81, 1 gene) and flavonoid 3′-monooxygenase (EC:1.14.14.82, 4 genes) genes were found to be involved in “flavone and flavonol biosynthesis” (ko00944) and were downregulated during the synthesis of luteolin and quercetin. The flavonol-3-O-glucoside L-rhamnosyltransferase (EC:2.4.1.159, 1 gene) was found in “flavone and flavonol biosynthesis” (ko00944) and was downregulated during the synthesis of rutin (Figure 6).

In addition, 2,7,4′-trihydroxyisoflavanone 4′-O-methyltransferase (EC:2.1.1.212, 1 gene) and isoflavone 3′-hydroxylase (1.14.14.88, 2 genes) were found to be involved in “isoflavonoid biosynthesis” (ko00943) and were upregulated during the synthesis of pseudobaptigenin and pratensein. The isoflavone 2′-hydroxylase (I2′H) is involved in isoflavonoid biosynthesis (ko00943) and is downregulated during the synthesis of 2′-hydroxybiochanin A 2′-hydroxygenistein (Figure 6).

### 3.9. Antifungal Activity Analyses

**Figure 8:**
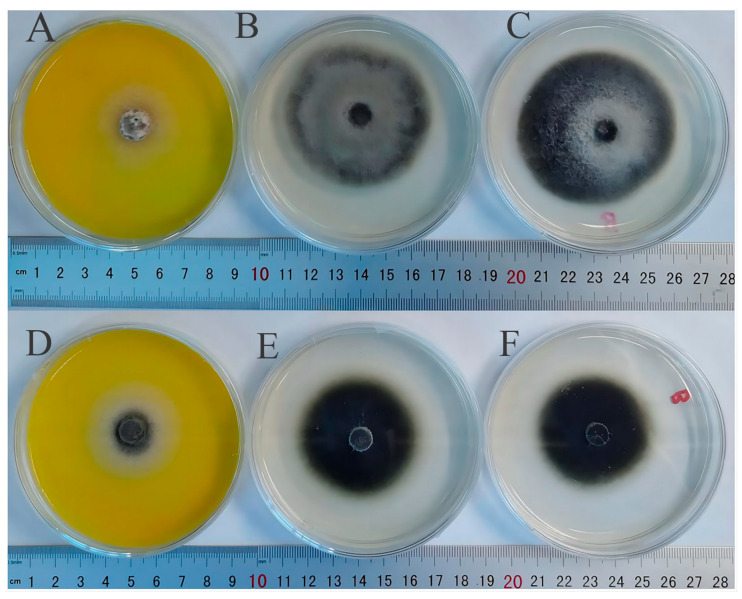
The growth of *A. tenuissima (Kunze)Wiltshire* and *M. oryzae* hyphae in treatment and CK media. (**A**) The treatment group of *Alternaria tenuissima (Kunze)Wiltshire*; (**B**) the CK group of *A. tenuissima (Kunze)Wiltshire*; (**C**) the blank group of *A. tenuissima (Kunze)Wiltshire*; (**D**) the treatment group of *M. oryzae*; (**E**) the CK group of *M. oryzae*; (**F**) the blank group of *M. oryzae*.

**Figure 9:**
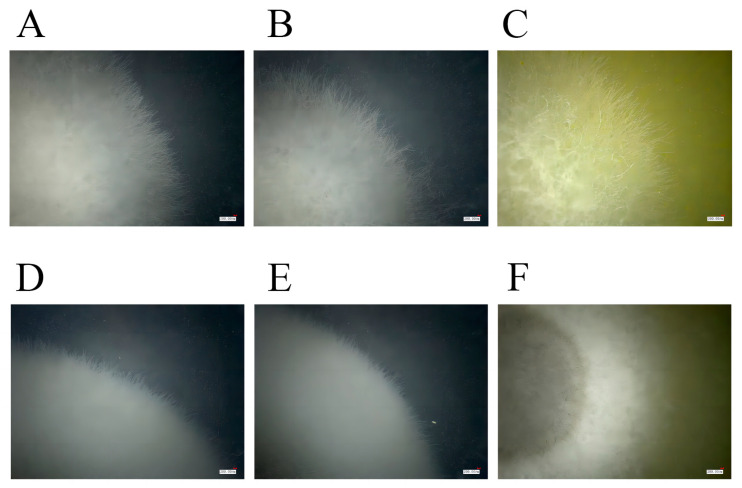
The growth of *A. tenuissima (Kunze)Wiltshire* and *M. oryzae* hyphae in blank, treatment, and CK media after 5 days. ((**A**): the treatment group of *A. tenuissima (Kunze)Wiltshire*; (**B**): the CK group of *A. tenuissima (Kunze)Wiltshire*; (**C**): the blank group of *A. tenuissima (Kunze)Wiltshire*; (**D**): the treatment group of *M. oryzae*; (**E**): the CK group of *M. oryzae*; (**F**): the blank group of *M. oryzae*).

The diameter, density, and color were recorded every day. With time, the fungal colony color in the experimental group became darker than those in the CK and blank groups, the diameter became longer than those in the CK and blank groups, and the density became higher than those in the CK and blank groups (Figure 8 and Figure 9).

After 7 days, the colony diameters of the two fungal treatment groups were significantly smaller than those of the CK group (Figure 8). The growth diameters of *A. tenuissima (Kunze)Wiltshire* and *M. oryzae* in the treatment group were obviously shorter than those in the CK group (Figures S5 and S6). The colony diameter had shown a significant difference (Table 3). The inhibition ratios were 42.16% for *A. tenuissima (Kunze)Wiltshire* and 37.22% for *M. oryzae* (Table 3).

As observed by super depth-of-field 3D microscopy, both the control group and blank group of two fungal strains showed dense growth and obvious accumulation. However, in the two fungal treatment groups, relatively few and sparse hyphae were distributed around the colony, and the accumulation phenomenon was not obvious (Figure 9). These results showed that the ethanol extract of fenugreek seeds has an inhibitory effect on *A. tenuissima (Kunze)Wiltshire* and *M. oryzae*.

## 4. Discussion

Although fenugreek plants have been cultured for many years and widely used as medicine in many countries, the components, especially the main medicinal components of the seeds, have not been fully elucidated. Previous studies have reported six saponins isolated from the methanol extract of fenugreek seeds from India; trigonelline and N-methylnicotinic acid in fenugreek seeds; flavonoids such as quercetin, luteolin, and vitexinin in whole fenugreek leaves; alkaloidal and glycosidal fractions in fenugreek leaves [19,20,21]; and the alkaloidal fraction, which is effective at reducing inflammation [22]. In this study, the global view of fenugreek seed components was obtained by metabolomic analysis. There were a total of 9499 metabolites identified in the positive ion mode, and 8043 metabolites were identified in the negative ion mode in metabolomic analysis. Among them, flavonoids, alkaloids, and steroid derivatives have been reported in previous studies [19,20,21] (Table S7). In addition, 151 fatty acyls and 70 prenol lipids were identified first in fenugreek seeds. The lipids and starches may accumulate with the development of fenugreek seeds.

The result of metabolome analysis showed the types of flavonoids that occurred most in the fenugreek seed. In total, 58 flavonoids were identified in our study. Among them, there were many flavonoids, and isoflavonoids were significantly upregulated according to the analysis of the different metabolites. The present study results showed that the contents of flavonoids and isoflavonoids increased in the later stages of seed development. One study identified flavonoid glycosides in fenugreek seeds, and the main flavones were apigenin adducts, followed by luteolin derivatives [5]. Another study also identified astragalin and apigenin-7-O-β-D-glucoside in fenugreek seeds by liquid chromatography–mass spectrometry (LC–MS) analysis [23]. Taken together, these studies showed that flavonoids and related derivatives may be the main secondary metabolites in fenugreek. In addition, in the present study, several metabolites, such as chrysoeriol, dihydrokaempferol, and sanguinarine, were identified in fenugreek for the first time, but they were not found in previous research. The metabolome has been shown to have a significant advantage in exploring plant components. Previously, transcriptome sequencing had been used to analyze the leaf, stem, and root tissues of fenugreek plants. Enzymes involved in the biosynthesis of several metabolites were found, and they were highly expressed in the fenugreek plants [14]. However, there is no research on the gene expression of these seeds. In the present study, a transcriptome sequencing analysis of fenugreek seeds was conducted, and 1079 DEGs were enriched in the “biosynthesis of other secondary metabolites”. Among them, the number of DEGs involved in “phenylpropanoid biosynthesis” and “flavone biosynthesis” was greatest. The flavonoids and alkaloids accumulated with the development of fenugreek seeds. This can further explain the synthesis mechanism of these metabolites.

Several studies have shown that flavonoids, isoflavonoids, and alkaloids have inhibitory effects on cancer cell proliferation to help cure many cancers and other important pharmacological effects [24,25]. Pratensein is an isoflavone extracted from *Radix Polygala* roots. One study reported that pratensein has anti-inflammatory and antioxidant effects [24]. Another study revealed that sanguinarine has inhibitory effect on the proliferation of nasopharyngeal carcinoma cells [25]. Although some studies have reported that fenugreek has pharmacological effects on some human diseases [24,25], few reports have comprehensively explored the medicinal compounds present in fenugreek. It was reported that flavones, isoflavones, steroidal saponins, and diosgenin in fenugreek were used in reproductive processes, lactation, estrogenic activity, and diabetes [26,27]. According to our transcriptome and metabolome analyses and previous reports, flavonoids, isoflavonoids, and alkaloids are thought to be important components in fenugreek fruits and should receive increased attention. The results of the present research showed that prstensein and sanguinarine were enriched in metabolic pathways. Additionally, pratensein was significantly upregulated in the KEGG synthetic pathway (map00943 isoflavonoid biosynthesis). Sanguinarine was significantly upregulated in the KEGG synthetic pathway (map00950, isoquinoline alkaloid biosynthesis). Thus, these metabolites may have research value for exploring the pharmacological properties of fenugreek.

The present study also combined metabolomic and transcriptomic analyses. The present study found that several metabolites and enzymes were enriched in the same metabolic pathways and were co-upregulated. For example, in isoflavonoid biosynthesis (ko00943), 2,7,4′-trihydroxyisoflavanone 4′-O-methyltransferase (HI4OMT) and isoflavone 3′-hydroxylase (CYP81E9) were significantly upregulated, and they catalyzed the conversion of genistein to biochanin A, which can ultimately be converted to pratensein. Isoflavone 3′-hydroxylase (CYP81E9) has activity on the isoflavonoid compounds 20-hydroxyformonetin, pseudobaptigenin, and daidzein [28]. It may be a key enzyme involved in the synthesis of flavonoids in fenugreek seeds. In the present research, the expression of pratensein and pseudobaptigenin was also significantly upregulated according to the metabolome DAM analysis. Previous research has confirmed that isoflavone 3′-hydroxylase is key for controlling the synthesis of pratensein and pseudobaptigenin. Therefore, the present study hypothesizes that the isoflavone 3′-hydroxylase gene is involved in the accumulation of pratensein and pseudobaptigenin after fenugreek plants grow older. These results provide key information for understanding biosynthesis in fenugreek seeds.

Flavonol synthase (FLS) is a key enzyme involved in the synthesis of flavonoids. It can catalyze the conversion of dihydroflavonols into flavonols [29]. It has an important effect on anthocyanin accumulation [30]. Many FLS genes have been cloned and identified in other plants [31]. In the present research, it was found that FLSs are involved in the synthesis of many flavonoids and were significantly upregulated according to the DEG analysis of the transcriptome. Thus, the FLS gene is related to many flavonoids, such as kaempferol [29]. DEG analyses in the present study showed that FLS is one of the key genes affecting the synthesis of flavonoids.

Chalcone synthase (CHS) is a key rate-limiting enzyme in flavonoid biosynthesis. It can convert chalcones into dihydroflavones [32]. According to our DEG analyses, the CHS gene was also significantly upregulated during the development of seeds according to the results of the transcriptome analysis. This difference is related to the synthesis of significantly upregulated metabolites, such as phlorizin and naringenin chalcone. Thus, these findings further prove that the CHS gene has an important effect on controlling flavonoid biosynthesis. In pear, it can resist black spot disease [33]. Thus, it is necessary to conduct further research on the function of these genes and enzymes in plants.

Many studies have confirmed that plant extracts have antibacterial and antifungal activities. One study revealed that the seeds of fenugreek plants have inhibitory effects on several drug-resistant bacteria [15]. Two phytopathogenic fungi were selected for the antifungal activity test. Finally, the present study found that the ethanol extract of fenugreek seeds has an obvious inhibitory effect on *A. tenuissima (Kunze) Wiltshire* and *M. oryzae*. Another study revealed that the methanol extract of fenugreek whole plant parts had an inhibitory effect on *Alternaria* sp. and *R. solani*, and the seed extract also had the strongest inhibitory effect [34]. It was inferred that the inhibitory effect on the growth of *A. tenuissima* and related fungi resulted from the main secondary metabolites in the fenugreek seeds. One study reported that aqueous extracts of fenugreek can increase resistance to rice blast disease [35]. The results of our research further confirmed that fenugreek has antifungal activity against the rice blast pathogen *M. oryzae*. Another research study found the tangeretin had effect on the inhibition of *M. oryzae* [36]. The tangeretin was also identified in the result of metabolome analysis (Table 2). Thus, it has further proved there are some antifungal activity metabolites in fenugreek.

## 5. Conclusions

This study found metabolite differences during the fenugreek seed development and related pathways of some important metabolites by metabolomic and transcriptomic analyses. The antifungal activity test validated fenugreek seeds had antifungal activity. These antifungal metabolites may have been found in metabolomic analysis, which need to be identified in further research.

## Figures and Tables

**Table 1:** Metabolites that were differentially accumulated in late seed development.

Compounds	Fold Change	Pathway
**Flavonoids**:		
3,7-Di-O-methylquercetin	36.862	map00944 Flavone and flavonol biosynthesis
Luteolin 7-O-β-D-diglucuronide	27.739	map00944 Flavone and flavonol biosynthesis
Apigenin	24.697	map00941 Flavonoid biosynthesis
Apigenin 7-O-β-D-glucoside	21.996	map00941 Flavonoid biosynthesis
Naringin	18.806	map00941 Flavonoid biosynthesis
Chrysoeriol	16.954	map00944 Flavone and flavonol biosynthesis
Rutin	16.662	map00944 Flavone and flavonol biosynthesis
Kaempferol 3-O-glucoside	14.202	map00944 Flavone and flavonol biosynthesis
Luteolin 7-O-β-D-glucoside	11.333	map00944 Flavone and flavonol biosynthesis
Dihydrokaempferol	8.712	map00941 Flavonoid biosynthesis
Luteolin 7-O-[β-D-glucuronosyl-(1->2)-β-D-glucuronide]-4′-O-β-D-glucuronide	8.27	map00944 Flavone and flavonol biosynthesis
Epigallocatechin 3-gallate	7.897	map04152 AMPK signaling pathway
Phlorizin	7.408	map00941 Flavonoid biosynthesis
Kaempferide	6.491	map00944 Flavone and flavonol biosynthesis
(+)-Catechin	2.936	map00941 Flavonoid biosynthesis
Naringenin	2.463	map00941 Flavonoid biosynthesis
**Isoflavonoids**:		
Pseudobaptigenin	36.652	map00941 Flavonoid biosynthesis
2′-Hydroxybiochanin A	30.126	map00943 Isoflavonoid biosynthesis
2′-Hydroxygenistein	19.097	map00943 Isoflavonoid biosynthesis
Pratensein	16.954	map00943 Isoflavonoid biosynthesis
**Alkaloids and derivatives**:		
Ecgonine methyl ester	24.183	map00960 Tropane, piperidine, and pyridine alkaloid biosynthesis
Sanguinarine	19.123	map00950 Isoquinoline alkaloid biosynthesis

**Table 2:** Correlation analysis of flavonoids, isoflavonoids, alkaloids, and their derivatives with DEGs.

Compound	Gene Name	Correlation Coefficients	*p*-Values
**Flavonoids:** Apigenin	legumin B	0.999397805	0.000000544
Chrysosplenol D	legumin B	0.999574004	0.000000272
Cosmosiin	legumin J	0.999622259	0.000000214
Resokaempherol	legumin J	0.999758896	0.0000000872
	seed linoleate 9S-lipoxygenase-3	0.9991967	0.000000968
Kaempferitrin	H/ACA ribonucleoprotein complex	0.999758896	0.0000000872
	subunit 4		
	HMG (high mobility group) box protein	0.9991967	0.000000968
	with ARID		
**Isoflavonoids:** 3,7-Di-O-methylquercetin	legumin J	0.999412712	0.000000517
Apigenin dimethylether;	aldehyde decarbonylase	0.999397805	0.000000544
Astragalin	legumin B	0.999583385	0.00000026
Tangeretin	UPSTREAM OF FLC protein	0.999001756	0.00000149
Pratensein	legumin J	0.999724414	0.000000114
Dehydroferreirin;	legumin J	0.999650167	0.000000184
**Alkaloids and derivatives:**			
Harmalol	(R)-mandelonitrile β-glucosyltransferase	0.999715273	0.000000122
(+/−)-6-Acetonyldihydrosanguinarine	tetratricopeptide repeat (TPR)-containing protein	0.99925841	0.000000825

**Table 3:** The inhibition rate of hyphal growth after 7 days.

Phytopathogenic Fungi	Treat (Diameter/cm)	CK (Diameter/cm)	Blank (Diameter/cm)	Inhibition Rate
*M. oryzae*	3.85 ± 0.18 b	6.13 ± 0.08 a	5.63 ± 0.63	37.22%
*A. tenuissima (Kunze)Wiltshire*	3.77 ± 0.28 b	6.52 ± 0.25 a	6.67 ± 0.16	42.16%

Note: the lowercase letters show significant difference across treatment and CK.

## Data Availability

The data that support the findings of this study are available from the corresponding author upon reasonable request.

## Supplementary Materials

The following supporting information can be downloaded at https://www.mdpi.com/article/10.3390/genes15030343/s1. Figure S1. Pie chart of secondary metabolites identified by metabolome. Figure S2. Gene annotation Venn diagram in transcriptome analysis. Figure S3. (A) Circos plots of DEG and DAM correlation analysis in positive ion mode. (B) Circos plots of DEG and DAM correlation analysis in negative ion mode. Figure S4. The growth of *Alternaria tenuissima (Kunze)Wiltshire* in medium of blank, late, and CK. Figure S5. The growth of *Magnaporthe oryzae* in medium of blank, late, and CK. Table S1. All identified metabolites in metabolome analysis. Table S2. The classification of metabolites in metabolome analysis. Table S3. KEGG enrichment of metabolites in metabolome analysis. Table S4. GO enrichment of DEGs in transcriptome analysis. Table S5. KEGG enrichment of DEGs in transcriptome analysis. Table S6. Integrated metabolome and transcriptome integrated analysis. Table S7. Types and references of metabolites identified in previous studies.
