# Supplementary material for: Metabolomic and Transcriptomic Analyses Reveal the Molecular Mechanism Underlying the Massive Accumulation of Secondary Metabolites in Fenugreek (Trigonella foenum-graecum L.) Seeds
Source: Genes (Basel). 2024 Mar 7;15(3):343. doi: 10.3390/genes15030343 (PMC10969933; doi:10.3390/genes15030343)
Supplement: Supplementary file 1 [file genes-15-00343-s001.zip › Table S7 .pdf]

Table S7. Types and references of metabolites identified in previous studies.

**Metabolite**	**Author and time**	**Reference**
① 26-O-beta-D-glucopyranosyl-(25S)-5 alpha-furostane-2 alpha,3 beta,22 zeta,26-tetraol 3-O-[beta-D-xylopyranosyl (1 --> 6)]-beta-D-glucopyranoside;<br>② 26-O-beta-D-glucopyranosyl-(25R)- 5 alpha-furostane-2 alpha,3 beta,22 zeta,26-tetraol 3-O-[beta-D-xylopyranosyl (1-->6)]-beta-D-glucopyranoside;<br>③ 26-O-beta-D-glucopyranosyl-(25R)-5 beta-furostane-3 beta,22 zeta,26-triol 3-O-[beta-D-xylopyranosyl (1 --> 6)]-beta-D-glucopyranoside;<br>④ 26-O-beta-D-glucopyranosyl-(25R)-5 beta-furostane-3 beta,22 zeta,26-triol 3-O-[beta-D-xylopyranosyl (1-->6)]-beta-D-glucopyranoside;<br>⑤ 26-O-beta-D-glucopyranosyl-(25S)-5 alpha-furostane-3 beta,22 zeta,26-triol 3-O-[alpha-L-rhamnopyranosyl(1 --> 2)]-beta-D-glucopyranoside;<br>⑥ 26-O-beta-D-glucopyranosyl-(25R)-5 alpha-furostane-3 beta,22 zeta,26-triol 3-O-[alpha-L-rhamnopyranosyl (1 --> 2)]-beta-D-glucopyranoside.	Yoshikawa, etc. 1997	[19]
Trigocoumarin	Parmar, etc. 1982	[21]
Apigenin adducts and Luteolin derivatives	Benayad, etc. 2014	[4]
Astragalin and Apigenin-7-O-beta-D-glucoside	Yang, etc.2022	[23]
