# Supplementary figures and images for: Metabolomic and Transcriptomic Analyses Reveal the Molecular Mechanism Underlying the Massive Accumulation of Secondary Metabolites in Fenugreek (Trigonella foenum-graecum L.) Seeds
Source: Genes (Basel). 2024 Mar 7;15(3):343. doi: 10.3390/genes15030343 (PMC10969933; doi:10.3390/genes15030343)

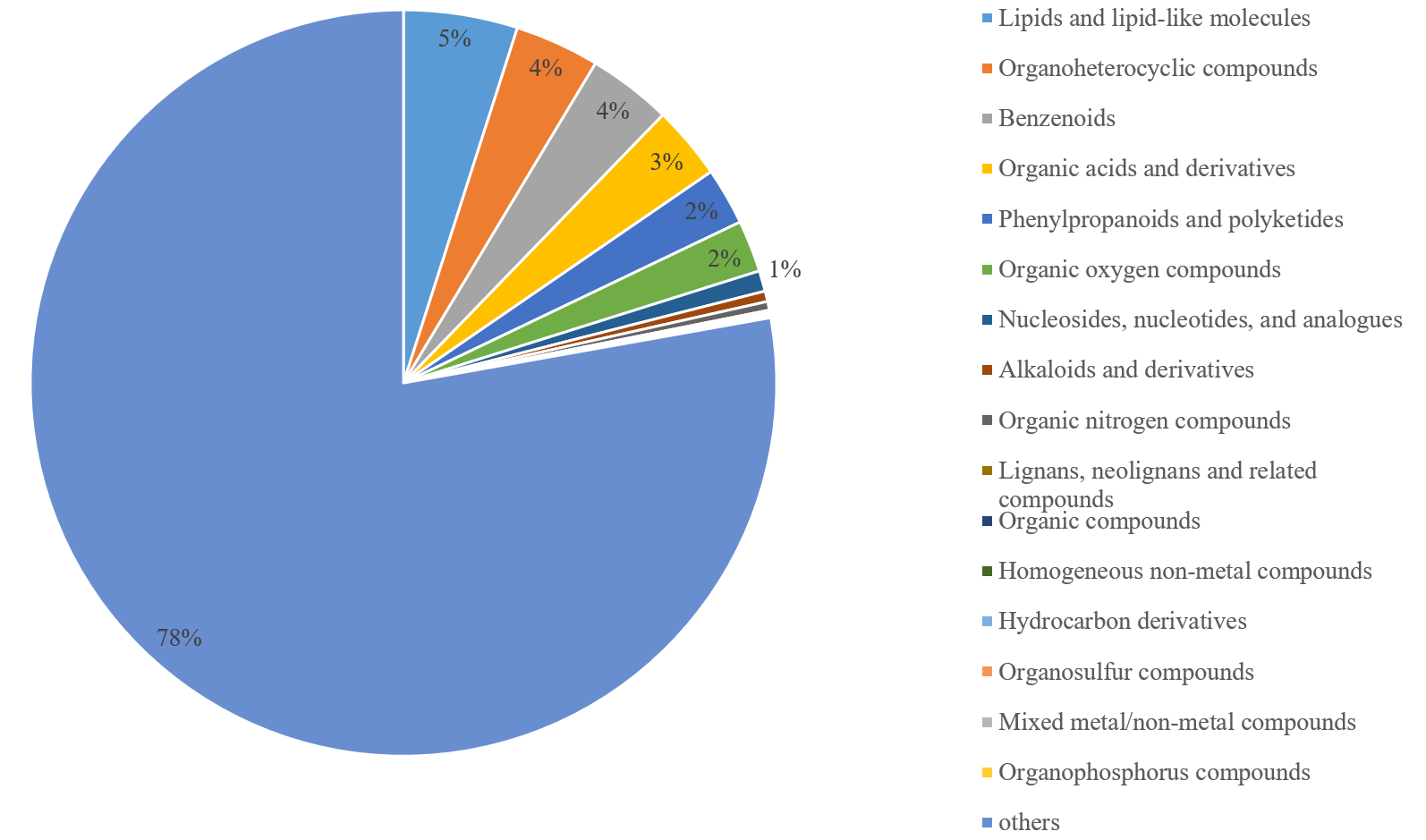

Supplement: Supplementary file 1 [file genes-15-00343-s001.zip › Figure S1.tif]

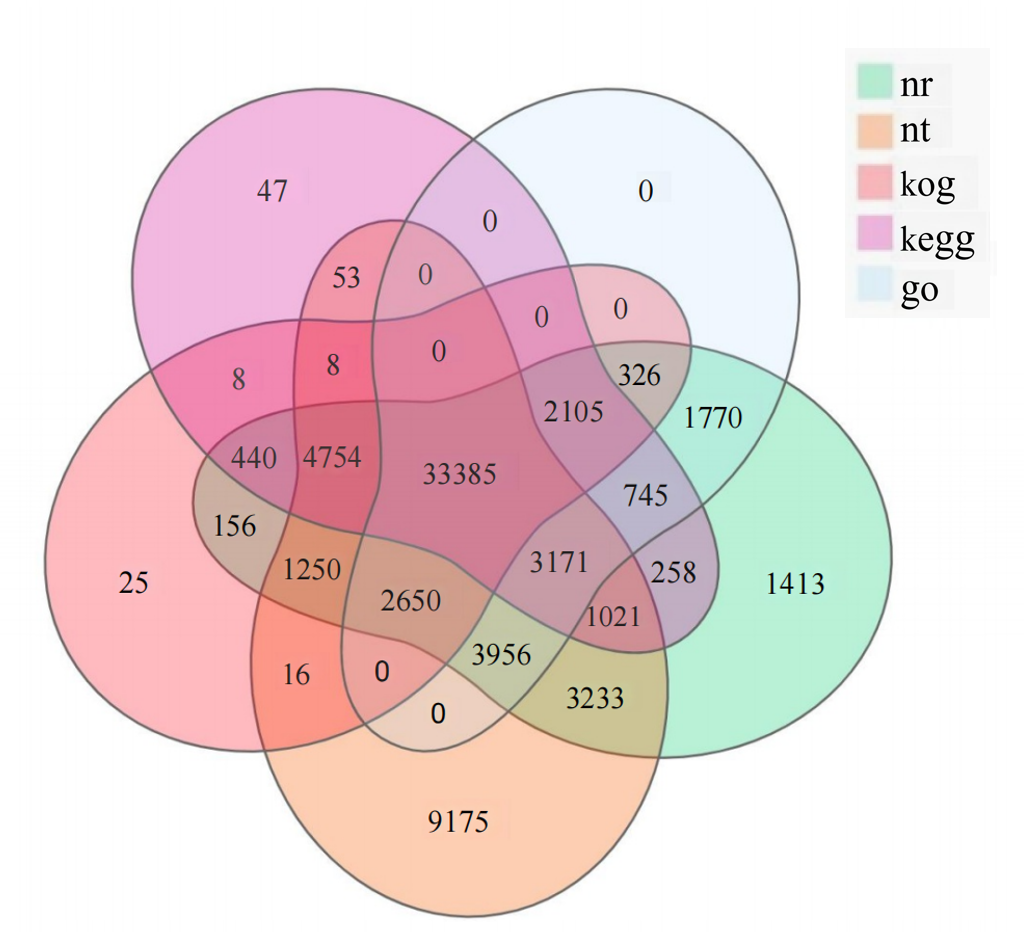

Supplement: Supplementary file 1 [file genes-15-00343-s001.zip › Figure S2.tif]

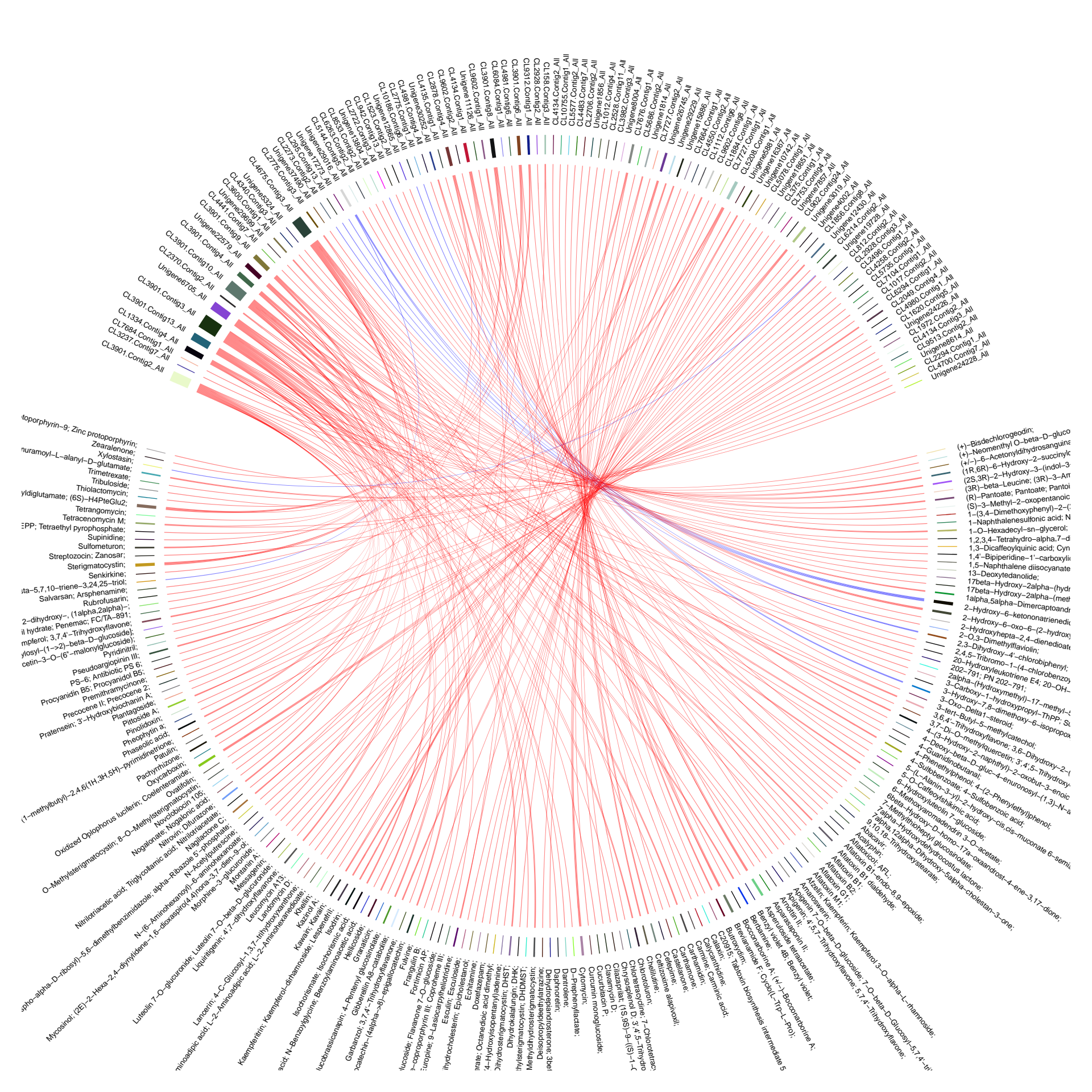

Supplement: Supplementary file 1 [file genes-15-00343-s001.zip › Figure S3A.pdf]

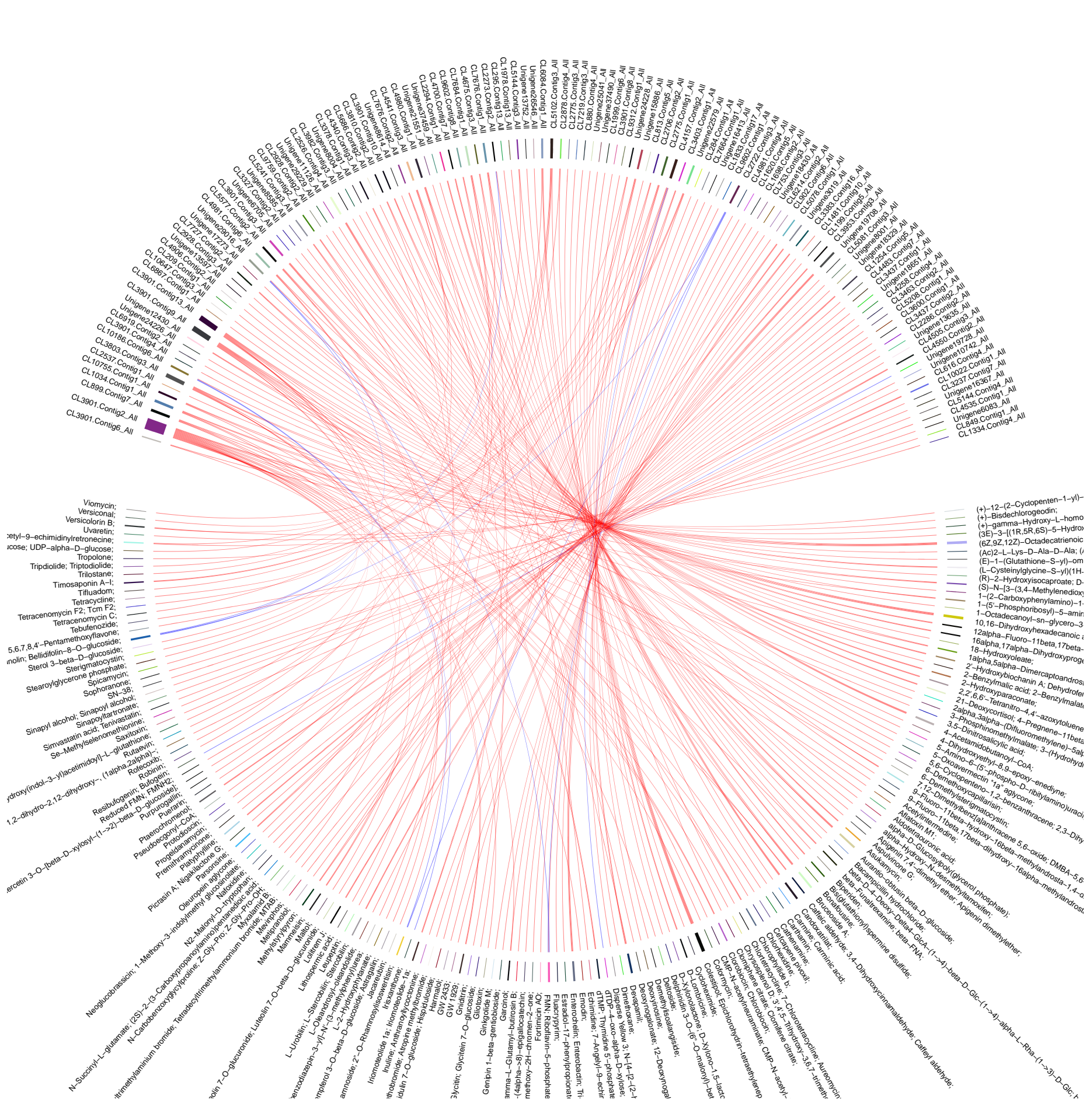

Supplement: Supplementary file 1 [file genes-15-00343-s001.zip › Figure S3B.pdf]

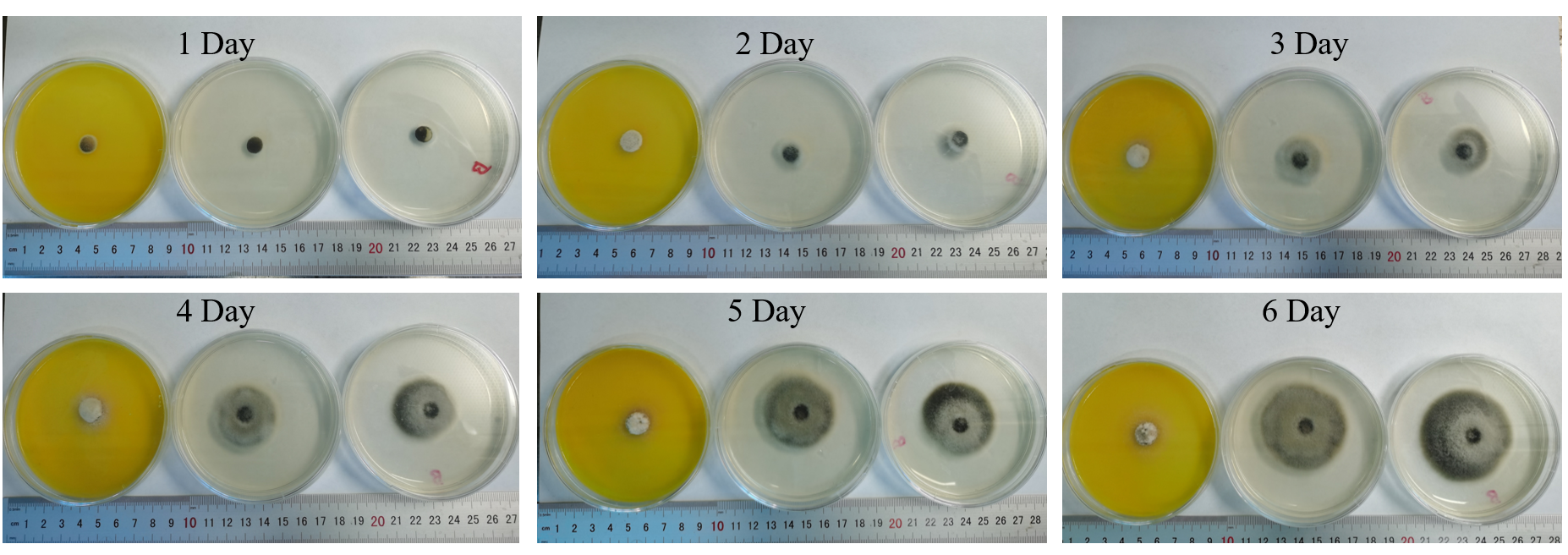

Supplement: Supplementary file 1 [file genes-15-00343-s001.zip › Figure S4.tif]

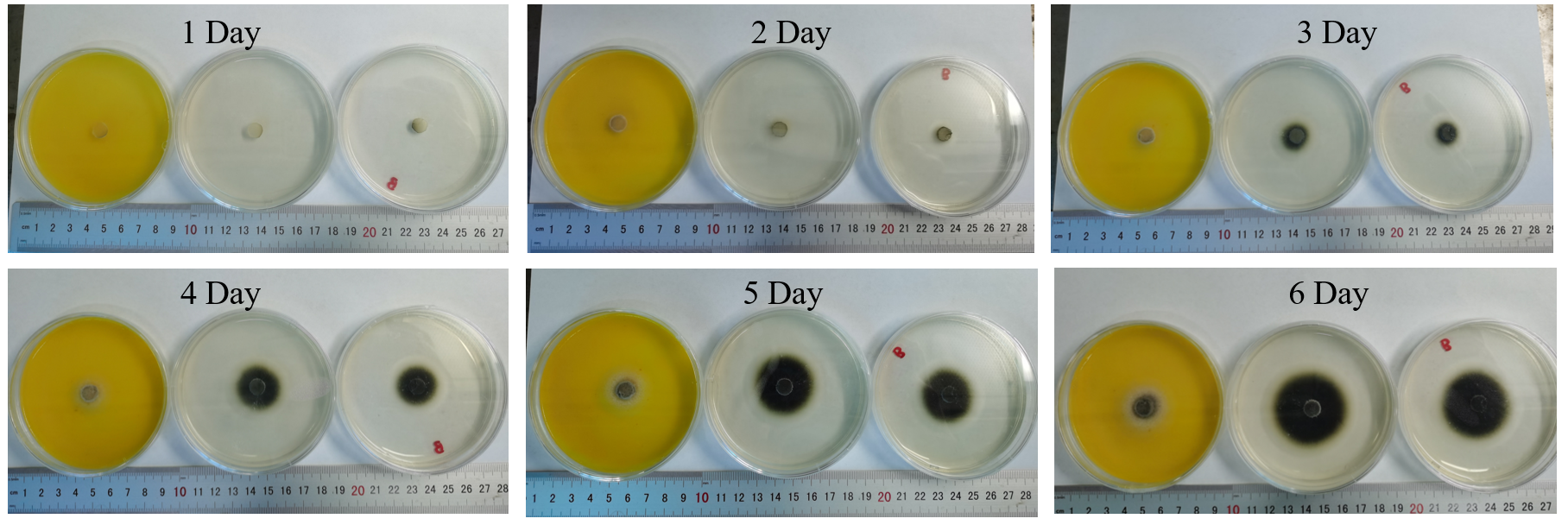

Supplement: Supplementary file 1 [file genes-15-00343-s001.zip › Figure S5.tif]
